# The association between deep learning approach and case based learning

**DOI:** 10.1186/s12909-019-1516-z

**Published:** 2019-04-11

**Authors:** Meenakshi Jhala, Jai Mathur

**Affiliations:** St George’s University of London, Cranmer Terrace, Tooting, London, SW17 0RE England, UK

## Abstract

Being medical students, and having experienced different learning approaches ourselves, here, we discuss and critically analyse the importance of the deep learning approach that Chonkar et al. have presented, alongside emphasizing Case Based Learning, and their roles in life long medical learning.

## Background

We would like to thank Chonkar et al. [1] on their recent article which critically analyses the importance of different learning approaches and the long-term outcomes on retention of knowledge in medical students. Following on from the concept of the deep learning approach, we understand that it can be time consuming; hence, 50.8% [1] of students had the strategic approach as their dominant one. Chonkar et al. [1] have notably shown encouragement towards approaches which enhance the deeper learning approaches. In this letter, we would like to put emphasis on the case based learning method which transitions onto problem based learning after the two pre-clinical years at St George’s University of London, alongside the outcome of the deep learning approach and different examinations.

## Main text

As medical students ourselves, we have witnessed the evolution of pedagogical methods, and based upon personal reflection, have found that understanding a subject at its core is more valuable than a surface, superficial understanding. As aforementioned, at St George’s, we have case based learning in the first 2 years of medical school which morphs into problem based learning from third year onwards. Several studies have shown the benefits of CBL in accordance to deep learning. A study by Mclean [2] believed that CBL “improves clinical performance and enhances clinical knowledge” which reinforces ours and Chonkar et al’s points [1] of using case based learning as a technique to promote the deep approach [2]. Comparisons between problem based learning and case based learning have also been made, with CBL named more “goal-oriented” [3]. This is due to the input of the instructor which allows for each student to have an equal contribution towards the diagnosis of a patient; they are “content experts” for each CBL session. Moreover, “high self-regulated learners” display increased ease with case based learning [4] and in our opinion, this may be due to their already developed skills and techniques to efficiently complete independent tasks. Thus, we believe that should the deep learning approach be emphasized more, case based learning would have a more fruitful outcome in lifelong learning. We personally enjoy CBL due to the engagement and theory application; a review by Williams [5] gives evidence of this overall.

Having experienced CBL first hand, we can agree that wanting to culminate a session with determining the diagnosis of a patient, and applying knowledge acquired through lectures set in the curriculum to reach that step, derives a form of satisfaction. It also grants a glimpse of why the pre-clinical years are important; motivation can far too often become lost, when only learning theory for 2 years continuously, without a practical element, and links to the occupation of clinical practice. Having short placements alongside our pre-clinical years puts everything into an occupational perspective and allows us to clinically apply theoretical knowledge we have in understanding pathologies; this enriches and fortifies our knowledge. We feel that this could be another complementary or adjuvant way to encourage the deep learning approach Chonkar et al. [1] speak about, alongside case based learning.

Chonkar et al. [1] discuss the effect of education before medical school having an influence in the way students learn, and the approaches they take, at medical school. This discourse is understandable, since in pre-medical school education, there is a homogenous, universalized objective of getting into medical school, which is the foundation for why the strategic learning approach is emphasized so heavily, as it is about having the relevant qualifications to be able to be accepted onto such a competitive course. However, this alters significantly when in medical school, because it is no longer necessarily about achieving a certain percentage at all times, but rather about choosing between several different career trajectories. We have found, with personal experience, many students realize their learning approach is not allowing them to gain maximum knowledge until far too late. Students who are “average” may believe their methods of surface or strategic studying are working and stay at that level; many students may simply have the target of passing examinations, so if that is ascertained, the task has been completed. Therefore, we second the point that the impetus must be on universities to reinforce certain learning approaches in medical school, which instils deep strategic revision methods in students.

Focusing more on the deep learning approach, as we know, medical school prepares us for future practice as health care professionals. Therefore, it is important that the examinations test our knowledge; the challenge herein is that it is not possible to have a holistic view on the extent of knowledge obtained by students. This is especially evident when students only apply the strategic or surface approach and do not have the opportunity to apply their knowledge to practical situations, because that depth of knowledge has not been taught and reinforced. Ergo, we appreciate the importance of the Objective Structured Clinical Examination (OSCE) as that allows for application of knowledge - without prompts, which are given in multiple choice question examinations. This is because, as future practicing clinicians, in tangible, real life situations, there will be no ‘hints’ given, hence showing the value of the deep approach for life-long learning, to treat patients. It is, thus, imperative to highlight to students that they are studying to make monumental differences in the lives of patients, rather than just pass examinations.

As we proceed onto clinical years, we have witnessed first-hand, the significance of having a robust understanding of medical concepts, as these are our foundations for the next step of investigations and management in patient care. This can be especially difficult as many patients have non-specific presenting complaints, such as in the case of ovarian cancer, ultimately leaving it down to a thorough investigation and deep clinical knowledge base that will allow differentiation between two or three prime differentials. Thus, this can be a challenge; if the broad and basic knowledge of specialties taught in the pre-clinical years is not up to standard, this engenders an obstacle towards being a competent doctor by the end of our course. Therefore, we believe, it is in the clinical years where the surface/strategic approach shifts to the deep approach, as many students are made aware of the gaps in their knowledge, and strive to actively “read up” in order to ascertain the level of knowledge needed for true medical practice. Case based learning and problem based learning can allow opportunities for students to modify and transition their learning methods; however, it is on placements, where the practical scenarios are present, that students truly appreciate the need of understanding and applying the deep learning approach, rather than surface learning. In our opinion, the deep learning approach has a direct correlation between the competency of a future doctor being able to provide holistic care.

## Conclusion

As the final aim is to treat patients, we believe case based learning is an outstanding start to transitioning students from more traditional and theoretical “text-book” methodologies, to practical scenarios. It allows students to think holistically, from the presenting complaint to the social aspect of the patient’s life, and we believe this is due to the effective deep learning approach. The advantages of deep understanding can then be seen to amplify further in the clinical years. We believe that, if students applied the deep learning approach from when they started medical school, their knowledge would reach higher echelons, clinical problem-solving ability would be enhanced, and there would be less reliance on “memorization” of diseases, and more on understanding how symptoms can constellate to diagnoses. To conclude, we understand that students adapt their learning approaches with different tasks, however as Chonkar et al. [1] have mentioned in their study, the deep learning approach is most effective and most relevant to the practice of a Doctor.

## Abbreviations

CBL: Case based learning
OSCE: Objective Structured Clinical Examination
