# Supplementary material for: The association between deep learning approach and case based learning
Source: BMC Med Educ. 2019 Apr 11;19:106. doi: 10.1186/s12909-019-1516-z (PMC6460538; doi:10.1186/s12909-019-1516-z)
Supplement: Related texts

# Reply: Response to “The association between deep learning approach and case based learning”

**Authors:** Kok Hian Tan, Sonali Prashant Chonkar, Li Xian Amy Tan, Bernard Chern

**Affiliations:** 3 0000 0000 8958 3388 grid.414963.d KK Women’s and Children’s Hospital, Singapore, Singapore; 4 0000 0004 0385 0924 grid.428397.3 Duke-NUS Medical School, Singapore, Singapore; 5 0000 0001 2180 6431 grid.4280.e Yong Loo Lin School of Medicine, National University of Singapore, Singapore, Singapore; 6 0000 0001 2224 0361 grid.59025.3b Lee Kong Chian School of Medicine, Nanyang Technological University, Singapore, Singapore

We note that Jhala & Mathur (both medical students) concurred with us on the importance of the deep learning approach for medical students. We agree that the Case Based Learning is useful for deep learning. However, due to time pressure and the format of knowledge and skills assessment, medical students often resort to superficial and strategic learning approaches to cope in medical schools.

Deep learning emphasizes understanding of concepts and relations, having an interest in ideas, application of knowledge and cognitively active learning behaviours [1]. Indeed, the essence of deep learning is real understanding and not memorisation of isolated facts. All medical schools need to play a part to ensure that the deep learning approach is rooted in their institutions. These involve evolving teaching and assessment methods to enhance deep learning for medical students. Assessment formats directly affect the choice of study strategies. If faculty are committed to preparing questions that require understanding and thinking skills, medical students will find that they cannot easily answer those questions with isolated facts that they have memorized. They will start learning differently. With better understanding, they can have better retention and effective application of the knowledge they acquired.

We are heartened that in many medical schools, pro-deep learning methods and assessments are increasingly utilised. These include Case Based Learning, Problem Based Learning, Team Based Learning and OSCE, which emphasise on understanding and applications in relation to the situation or scenario [6]. Forming a vibrant academic medicine culture with the healthcare system, where medical students can actively interact and learn from senior and also junior physicians in real life cases, is another excellent way for better teaching and deep learning [7].

It is important to note that learning methods should be holistic and multi-pronged. While basic facts and standard management to medical issues and problems can be managed through superficial and strategic learning approaches, real understanding with optimal retention of knowledge as well as optimal application appropriately customised to specific situations, require the deep learning approach. Not surprisingly, superficial learning assessment measures like non-scenario based multiple-choice questions, that test recall of facts, are increasingly being supplemented by pro-deep learning methods.

There is a need to enhance self-awareness of learning approaches so that the deep learning approach can be more consciously utilised as the predominant approach. Understanding of these learning approaches can be introduced in early medical student years to optimise learning for medical students.
